# Acute pericarditis due to pegylated interferon alpha therapy for chronic HCV hepatitis - Case report

**DOI:** 10.1186/1471-230X-11-30

**Published:** 2011-03-31

**Authors:** Cristina Popescu, Victoria Arama, Smaranda Gliga

**Affiliations:** 1 'Matei Bals' National Institute of Infectious Diseases, University of Medicine and Pharmacy 'Carol Davila' Bucharest, Grozovici 1, sector 2, Romania

## Abstract

**Background:**

Cardio toxicity due to interferon therapy was reported only in small case series or case reports. The most frequent cardiac adverse effects related to interferon are arrhythmias and ischemic manifestations. The cardiomyopathy and pericarditis are rare but can be life threatening. The predisposing factors for interferon cardio toxicity were described only for ischemic manifestations and arrhythmias.

**Case presentation:**

The authors report a case of pericarditis due to alpha interferon therapy for chronic hepatitis C, in a young woman without previous cardiac pathology. The clinical manifestations started during the 7-th month of interferon treatment. The cessation of interferon was necessary. After interferon discontinuation the patient recovered, with complete resolution of pericarditis. The patient scored 9 points on the Naranjo ADR probability scale, indicating a very probable association between pericarditis and interferon administration.

**Conclusion:**

If a patient receiving interferon therapy complains of chest pain of sudden onset, a cardiac ultrasound should be performed in order to rule out pericarditis. We point out the possibility of an infrequent but severe adverse effect of interferon therapy.

## Background

Pegylated interferon plus ribavirin represents the gold standard therapy for hepatitis C virus (HCV) but various side effects may occur, limiting its efficacy [1,2]. Although some of these adverse effects are rarely reported, their severity can be higher than expected, sometimes leading to a life-threatening event. The diversity of the described adverse effects may increase because of the high number of patients who receive treatment for HCV hepatitis. Their monitoring and reporting is important in order to facilitate subsequent recognition of similar cases.

We report a case of acute pericarditis without tamponade which was correlated with pegylated interferon alpha 2a treatment for chronic HCV hepatitis.

## Case Presentation

A 38-year-old white female, without previous cardiac pathology, was being monitored and treated for chronic HCV hepatitis, genotype I virus. She was started on a combination treatment with peginterferon alpha 2a (180 μg weekly) and ribavirin (1000 mg daily, according to her body weight). The patient had complete early virologic response (HCV-RNA was undetectable at 12 weeks of treatment). In the first seven months of treatment no severe side effects (hematologic, endocrine, ophthalmic or autoimmune) were observed.

In the 7^th ^month of treatment the patient accused chest pain, dry cough, fatigue, dyspnea, without fever and she presented herself to the emergency room.

On physical examination we found: tachypnea (22-24/min), tachycardia (96/min), a blood pressure of 100/70 mmHg with pulmonary and abdominal examination within the normal limits. Cardiac examination revealed a third heart sound over the precordium as well as muffled cardiac sounds. The pulmonary X-ray was normal and the ECG showed no specific repolarisation abnormalities. Trans-thoracic cardiac ultrasonography revealed a pericardial effusion without tamponade. Laboratory data revealed: mild leukopenia, anemia, thrombocytopenia (which didn't need reduction of interferon or ribavirin doses), minor biological inflammatory syndrome (CRP - 17 mg/l). The hepatic enzymes were normal, HCV-RNA was undetectable, serological tests for viruses and bacteria which could have explained the pericarditis were negative: ECHO, Coxsackie, adenovirus, influenza virus, parainfluenza virus, EBV, CMV, HIV, Mycoplasma pneumoniae, Chlamydia pneumoniae, Rickettsia spp, Legionella pneumophila, Borrelia burgdorferii. Serological tests for viruses remained negative for the following 2 weeks. Quantiferon TB gold was negative. The patient didn't receive antimycobacterial medication. The ASO titer was in the normal range. Autoimmune tests were performed, with negative anti-nuclear factor, anti-DNA antibodies, p ANCA, c ANCA, anti-mitochondrial antibody, anti-Ro and anti-La antibodies and negative cryoglobulinemia. Thyroid function tests were normal, and anti tyreo-peroxidase antibodies were absent.

The antiviral medication was stopped and the patient received ibuprofen. The symptoms disappeared and the pericardial effusion resolved in 15 days.

Because the patient was infected with genotype 1 virus and detection of HCV-RNA at 4 weeks of treatment was not performed (in order to confirm a rapid virological response), the antiviral treatment was restarted only with pegylated interferon. After the first dose of interferon administration symptoms reappeared. The echocardiography showed an increase of pericardial fluid (at 24 hours after interferon administration). The antiviral medication was stopped again with rapid recovery.

Six months after discontinuation of treatment, the HCV-RNA was undetectable (the patient had sustained virologic response).

The correlation of pericarditis with interferon administration was appreciated, for the presented case using the Naranjo ADR probability scale, which summed up 9 points, indicating a very probable association (table 1) [3].

**Table 1:** NARANJO ADR PROBABILITY SCALE calculated for our case summed up 9 points, indicating a very probable association between acute pericarditis and interferon treatment

Question	Response	Points
**Are there previous conclusion reports on this reaction?**	Yes	+ 1
**Did the adverse event appear after the suspect drug was administered?**	Yes	+2
**Did the AR improve when the drug was discontinued or a specific antagonist was administered?**	Yes	+ 1
**Did the AR reappear when drug was readministered?**	Yes	+ 2
**Are there alternate causes [other than the drug] that could solely have caused the reaction?**	No	+2
**Did the reaction reappear when a placebo was given?**	don't know	0
**Was the drug detected in the blood [or other fluids] in a concentration known to be toxic?**	No	0
**Was the reaction more severe when the dose was increased, or less severe when the dose was decreased?**	don't know	0
**Did the patient have a similar reaction to the same or similar drugs in any previous exposure?**	No	0
**Was the adverse event confirmed by objective evidence?**	Yes	+ 1
**Total**		9

## Discussion

Interferon plays an essential role in the mechanisms of defense against infections, being produced by a variety of cells. Until now two types of interferon have been identified: type I (alpha and beta interferon), which blocks the translation and viral replication and type II (gamma interferon), which initiates the synthesis of chemical substances with antiviral properties [4]. On top of the positive effects, the administration of interferon can be associated with many unwanted adverse effects because of the destruction of uninfected cells. The prevalence of life threatening complications during the interferon treatment is less then 1%. The cardio toxicity of interferon has been even rarely reported, only in small series of patients, and often just in isolated cases [5]. Among the three types of interferon, the alpha interferon is the most cardio toxic, followed by beta and gamma interferon.

The mechanism through which interferon is cardio toxic has not been clearly demonstrated [6,7], but it is presumed that at least two factors are implicated: the deterioration of endothelial cells inducing the overlay of immune complexes at this level; and stimulation of TNF alpha, IL 2, IL 6, IL 1 release, that influence the vasopressor response.

The most common cardio toxic clinical effects of interferon are: arrhythmias- 58%, acute coronary syndrome- 21%, cardiomiopathies-12% and other manifestations - 9% (including pericarditis) [5]. There are no described predisposing factors for interferon cardio toxicity.

Acute pericarditis is determined by a multitude of causes: infectious, neoplastic, uremia, autoimmune, traumatic, drug induced. Concerning drug toxicity, there have been reported cases of acute pericarditis after the administration of: hydralazine, procainamide, izoniazid, phenylbutazone, dantrolene, doxorubicin, penicillin, these situations being extremely rare. Interferon is not cited by the ESC Guidelines on the Diagnosis and Management of Pericardial Diseases [8] as one of the causes of drug induced pericarditis.

Acute pericarditis, as a consequence of interferon treatment is extremely rare. So far, very few cases of pericarditis related to administration of interferon have been reported; some of these patients received interferon for neoplastic diseases such as melanoma or acute leukemia [9,10]. The role of ribavirin in the pathogenesis of pericarditis could be considered. In our case, we restarted only interferon therapy (after two weeks of discontinuation), which concluded in the reappearance of pericardial effusion.

Six cases of pericarditis due to interferon alpha have been reported in patients with chronic HCV hepatitis. Three of these patients had an underlying pathology that could explain the pericarditis: Sikole et al. reported a case of pericarditis among hemodialysis patients receiving interferon for hepatitis C [11], Suarez A et al described a case of pericarditis in a patient with cryoglobulinemia [12], Boonen et al. reported a case of pericarditis during the treatment of chronic HCV hepatitis as part of a lupus-like syndrome, with clinical manifestations of auto-immune pathologies [13]. Recently Kazuahi Nishio et al described a case of pericarditis related to pegylated interferon therapy for HCV hepatitis. This patient had positive anti-DNA and anti-ds DNA IgM antibodies [14]. The case of pericarditis reported by Gressens B et al in conjunction with the administration of interferon occurred four months after stopping the treatment [15].

We are reporting a very rare adverse effect of pegylated interferon treatment- acute toxic pericarditis, this being the second case observed in a patient with no underlying pathology, described in the medical literature. The first case was reported by Wisniewski B et al; pericarditis occurred in this case after the first dose of interferon [16].

In summary, we report a patient without any history of cardiac disease who developed pericarditis after interferon treatment. Reappearance of symptoms when the antiviral treatment was restarted indicates a strong correlation between drug and adverse effects [3]. The effect of the virus itself is quite unlikely (undetectable HCV-RNA). The clinical and biological markers of a possible associated autoimmune pathology were absent. We excluded a viral or bacterial (including tuberculosis) etiology of pericarditis.

## Conclusions

We consider that in a patient receiving interferon, pericarditis must be evoked if the patient accuses sudden chest pain. It is of most importance that a cardiac ultrasound should be performed in a situation like this.

## Abbreviations

HCV: hepatitis C virus; HCV-RNA: hepatitis C virus - ribonucleic acid; ECG: electrocardiogram; CRP: C reactive protein; EBV: Epstein Barr Virus; CMV: cytomegalovirus; HIV: human immunodeficiency virus; DNA: deoxyribonucleic acid; c ANCA: classical antineutrophil cytoplasmic antibodies; p ANCA: protoplasmic-staining antineutrophil cytoplasmic antibodies; ADR: adverse drug reaction; TNF: tumor necrosis factor; IL6: interleukin 6; IL2: interleukin 2; IL1: interleukin 1; ESC: European Society of Cardiology

## Competing interests

The authors declare that they have no competing interests.

1. *Financial competing interests*

• In the past five years I did not received reimbursements, fees, funding, or salary from an organization that may in any way gain or lose financially from the publication of this manuscript, either now or in the future.

• I do not hold any stocks or shares in an organization that may in any way gain or lose financially from the publication of this manuscript, either now or in the future.

• I do not hold and I am not currently applying for any patents relating to the content of the manuscript. I have not received reimbursements, fees, funding, or salary from an organization that holds or has applied for patents relating to the content of the manuscript.

• I have not any other financial competing interests.

2. *Non-financial competing interests*

There are not any non-financial competing interests (political, personal, religious, ideological, academic, intellectual, commercial or any other) to declare in relation to this manuscript.

## Authors' contributions

CP and VA contributed equally to this work; CP, VA, SG performed a review of the literature; CP and VA take care of the patient; CP, VA have been involved in drafting the manuscript and revising it critically; CP, VA and SG wrote and revised the paper before submission.

All authors read and approved the final manuscript

## Authors' information

CP - assistant professor at Carol Davila University of Medicine in Bucharest, MD from 1995, PhD from 2005, infectious diseases specialist from 2000, with a postdoctoral bursary supported by the Sectoral Operational Programme - Human Resources Development financed from the European Social Fund and by the Romanian Government. CP is the co-author of 4 books, more than 50 studies communicated to national and international medical conferences.

AV - associated professor at Carol Davila University of Medicine in Bucharest, MD from 1987, PhD from 1999, infectious diseases specialist from 1993. AV is the head of Third Department in Matei Bals National Institute of Infectious Diseases. VA is the author of more than 10 medical books and she published more than 100 medical papers, and communicated more than 100 medical studies.

SG - MD from 2009, resident physician in infectious diseases

## Consent

Written informed consent was obtained from the patient for publication of this case report and any accompanying images. A copy of the written consent is available for review by the Editor-in-Chief of this journal.

## Pre-publication history

The pre-publication history for this paper can be accessed here:

http://www.biomedcentral.com/1471-230X/11/30/prepub
